# Longitudinal Reciprocal Effects of Physical Exercise, Executive Function, and Subjective Well-Being: A Three-Wave Random-Intercept Cross-Lagged Panel Model in Chinese Minority College Students

**DOI:** 10.3390/bs15070865

**Published:** 2025-06-26

**Authors:** Xueyan Bai, Lin Yang

**Affiliations:** School of Journalism and New Media, Xi’an Jiaotong University, Xi’an 710049, China

**Keywords:** physical exercise, executive function, subjective well-being, longitudinal study, minority students

## Abstract

Objective: This study investigates the longitudinal reciprocal relationships among physical exercise (PE), executive function (EF), and subjective well-being (SWB) in ethnic minority college students in China, with the aim of providing theoretical and practical guidance for their psychological and cognitive development. Method: A three-wave longitudinal design was employed over a nine-month period to collect data on PE, EF, and SWB from 482 ethnic minority college students in Shaanxi Province, China (M age = 20.3 years, 63% female). Data were analyzed using a random-intercept cross-lagged panel model (RI-CLPM), and multigroup analysis (MGA) was used to examine the moderating effects of gender, family ethnic composition, and residential area. Results: The study revealed significant positive correlations among PE, EF, and SWB at the between-person level. At the within-person level, the variables exhibited temporal stability, with earlier levels significantly predicting later levels, indicating cumulative effects. Key cross-lagged analyses unveiled significant dynamic reciprocal relationships among the three variables: earlier PE positively predicted subsequent EF and SWB, earlier EF positively predicted subsequent PE and SWB, and earlier SWB positively predicted subsequent EF. Importantly, these dynamic pathways and overall relationships were consistent across gender, family structure, and residential area, indicating robustness within the studied population. Conclusion: This study highlights the dynamic, reciprocal relationships among PE, EF, and SWB. Consequently, promoting physical activity and culturally sensitive interventions for ethnic minority college students is crucial for enhancing their psychological resilience and well-being. This research offers valuable insights for policymakers and educators.

## 1. Introduction

### 1.1. Background: Subjective Well-Being and Unique Challenges of Ethnic Minority College Students

Against the backdrop of globalization and the expansion of higher education, the subjective well-being (SWB) of Chinese ethnic minority college students faces unique challenges. SWB not only serves as a crucial indicator of their adaptation but profoundly reflects the psychological interplay under acculturative stress (Kessler et al., 2009; Yang et al., 2021). However, the significant Western-centric bias in existing SWB research (Carella et al., 2022; Reyes-García et al., 2021; Wu & Lee, 2022) leaves the SWB mechanisms of this sizable population, bearing complex cultural integration challenges, largely unexplored. This study focuses on Chinese ethnic minority college students (including Uyghur, Tibetan, Zhuang, Hui, and others), aiming to investigate how physical exercise (PE) and executive function (EF) influence their SWB and to examine the dynamic relationships among these three constructs. The goal is to provide a fresh perspective for the field of cross-cultural psychology regarding the adaptation mechanisms of ethnic minority college students, to offer targeted intervention strategies for enhancing their SWB, and to promote the development and application of relevant theories in diverse cultural contexts.

Within an educational and social system predominantly shaped by Han culture, Chinese ethnic minority college students commonly experience multiple burdens, including linguistic and cultural adaptation, economic and academic pressures, and the “dual cultural identity tension” (Chia & Hruschka, 2023; Gao & Liu, 2021; Gustafsson & Shi, 2003; Yao et al., 2023; Zhou, 2000). Preliminary research has cautioned that the SWB levels of Chinese ethnic minority college students are concerning (e.g., lower life satisfaction, higher prevalence of negative emotions) (Wei et al., 2010; Zhao & Hu, 2024), which may not only hinder their academic achievement and social integration but pose potential risks to their long-term development (L. Li et al., 2012; Yao & Yang, 2017). Therefore, an in-depth analysis of the key factors influencing the SWB of this population and their intrinsic mechanisms of action is of pressing practical and theoretical significance for developing effective intervention strategies and enriching cross-cultural psychological adaptation theories. Based on this, this study aims to explore the relationship among PE, EF, and SWB among Chinese ethnic minority university students. To further address the limitations in current research, this study uniquely employs a longitudinal design to investigate the dynamic relationships between PE, EF, and SWB specifically within Chinese ethnic minority college students while also considering the moderating effects of demographic factors. This approach aims to provide a more comprehensive understanding of SWB in this understudied population.

### 1.2. Potential Role of Physical Exercise and Mediating Mechanism of Executive Function

Against this backdrop, PE has garnered considerable attention as a cost-effective and readily implementable positive intervention owing to its potential to enhance the SWB of ethnic minority college students (Grasdalsmoen et al., 2020; Smith & Merwin, 2021). Self-determination theory (SDT) and social cognitive theory (SCT) provide key theoretical frameworks for understanding this phenomenon. Specifically, within the SDT framework, the autonomy experienced by ethnic minority college students in PE may manifest as choosing activities that align with their cultural backgrounds (e.g., sports with ethnic characteristics), thereby cultivating a sense of independence and control. Competence can be enhanced by mastering new skills or achieving personal fitness goals, thus boosting feelings of accomplishment and self-confidence. Relatedness can be strengthened by participating in group activities or gaining social support within exercise communities, thereby fostering a sense of belonging and connection. These factors not only directly enhance SWB but potentially alleviate feelings of loneliness and social isolation that may arise during cultural adaptation, which would align with the tenets of social support theory (Shannon et al., 2019). From a grounding in SCT, the enhanced self-efficacy derived from PE empowers students to cope with acculturative stress, such as discrimination or academic challenges, thereby bolstering their confidence to overcome adversity. This sense of mastery can be transferred to other areas of life, promoting overall SWB (Vasconcellos et al., 2020). It is worth noting that, in the context of cultural adaptation, PE may offer a unique source of cultural identity and belonging for ethnic minority college students, an aspect that has received limited attention in previous research. Existing studies indicate that for ethnic minority college students facing acculturative stress, PE is not only beneficial for physical and mental health but for alleviating cultural conflicts by promoting social interaction and reinforcing cultural identity, thereby becoming an effective means of enhancing their SWB (Beauchamp et al., 2019; Dai & Menhas, 2020; Schunk & DiBenedetto, 2020). The broaden-and-build theory of positive emotions further suggests that the enhancement of SWB may, in turn, increase the motivation to engage in PE, implying a dynamic and mutually reinforcing relationship between PE and SWB (Ghosh et al., 2024; Johnson et al., 2021; Sommerfeldt et al., 2019; St-Pierre & Richard, 2020).

Within this interplay, EF—encompassing higher-order cognitive regulation abilities such as working memory, inhibitory control, and cognitive flexibility (Doebel, 2020; Nejati et al., 2020)—is considered a key bridge connecting PE and SWB. PE can improve EF through pathways such as promoting the release of brain-derived neurotrophic factor (BDNF) and optimizing prefrontal cortex function (Zhang et al., 2024). For ethnic minority college students who often need to switch between different cultural frameworks and language systems and face higher cognitive regulation demands, the effective functioning of EF is crucial for managing the emotional distress caused by cultural conflicts and adapting to complex academic and social environments. Therefore, an in-depth understanding of the mechanisms of action of EF in this population is essential (Jie, 2024; Whitley et al., 2018). Although existing research has paid attention to the importance of EFs, empirical studies specifically targeting EF in this population, and its dynamic associations with PE and SWB, are still severely lacking (Liu & Yu, 2015; Zhang et al., 2021; Zhang et al., 2022), constituting a significant gap in current research. Some studies have also suggested that SWB may, in turn, influence EF and EP (Jang et al., 2020; Marshall et al., 2020; Pellegrini-Laplagne et al., 2023; Tan et al., 2020), further pointing to a complex interactive relationship among the three.

### 1.3. Research Questions, Hypotheses, and Analytical Approach

PE, EF, and SWB are of significant importance for individual development. However, existing research has several limitations: (1) the predominant use of cross-sectional designs hinders the elucidation of dynamic relationships among variables; (2) there is a scarcity of in-depth investigations into the dynamic reciprocal relationships among PE, EF, and SWB; and (3) limited attention has been paid to ethnic minority college students, particularly lacking exploration of how their unique sociocultural backgrounds influence their SWB.

To address these gaps, the present study proposes the following measures: (1) employing three-wave longitudinal data and utilizing the random-intercept cross-lagged panel model (RI-CLPM) (Kojima et al., 2021) to comprehensively examine the dynamic bidirectional relationships between PE, EF, and SWB among Chinese ethnic minority college students. Longitudinal studies enable tracking individual changes over time, revealing causal associations between variables. The RI-CLPM allows for simultaneous examination of between-person differences and within-person variations, providing a comprehensive analysis of variable relationships; (2) focusing on Chinese ethnic minority college students to investigate the relationships between PE, EF, and SWB within this population and employing multiple group analysis (MGA) to explore the moderating roles of demographic variables such as gender, family ethnicity, and residential location, thereby revealing the influence of cultural and social factors on these relationships. MGA can effectively examine the differences in variable relationships between different groups, thereby providing a more comprehensive understanding of the influencing factors of SWB of ethnic minority college students. Social gender was included as a moderator to account for the potential impact of gender roles on PE and EF. Family ethnicity was considered a crucial moderator because of culturally specific values influencing well-being. Residential location Was examined as a moderator reflecting differential access to resources and social support.

Based on the aforementioned theoretical analysis and literature review, this study proposes the following hypotheses:

**Hypothesis** **1.** *At the between-person level, there is a significant positive correlation among PE, EF, and SWB.*

**Hypothesis** **2.** *At the within-person level, there are dynamic reciprocal predictive relationships between PE, EF, and SWB.*

**Hypothesis** **3.** *Gender, family ethnicity, and residential location moderate the relationships between PE, EF, and SWB.*

### 1.4. Implications

This study is expected to provide valuable insights into the dynamic interplay between PE, EF, and SWB among Chinese ethnic minority college students. The findings will offer a scientific basis for developing targeted intervention strategies aimed at enhancing their SWB, particularly those leveraging PE and EF training. Furthermore, this research contributes empirical evidence and theoretical perspectives from a Chinese context, enriching the global understanding of psychological resilience and positive development in vulnerable youth within multicultural environments.

## 2. Methods

### 2.1. Participants

This study employed a three-wave longitudinal design over approximately one year to examine the dynamic relationships among PE, EF, and SWB) in a sample of Chinese ethnic minority college students. Participants were 482 students (M age = 20.3 years, SD = 1.42; 63% female) from Shaanxi Province. Participants were recruited using stratified random sampling from universities with higher proportions of ethnic minority students. Ethnic minority status was confirmed through self-report and university records. The sample primarily consisted of Uyghur (35%), Hui (30%), Tibetan (20%), Mongolian (10%), and other ethnic minority students (5%).

Data were primarily collected via online questionnaires on the Wenjuanxing platform, with most participants completing them in self-selected, private environments (e.g., homes, dormitories). To improve response rates, the research team also collaborated with some universities to administer the questionnaires in classrooms. For students completing questionnaires in classrooms, adequate spacing was ensured, and the importance of independent completion was emphasized to avoid mutual influence. Proctors were present to supervise students, who were instructed to remain quiet and avoid communication during questionnaire administration. To ensure data security, SSL encryption technology was used to encrypt data transmission. All questionnaire data were stored in secure electronic systems with strict access controls to prevent unauthorized access.

To ensure data quality, the research team implemented several measures: (1) detailed instructions at the beginning of the questionnaire; (2) required questions; (3) exclusion of questionnaires completed in under 5 min (determined by pilot data and expert opinion); and (4) exclusion of questionnaires with obvious response patterns indicative of random or inattentive responding. For remaining item-level missing data in valid questionnaires, full information maximum likelihood (FIML) estimation was used during structural equation modeling, a recommended approach for longitudinal studies. Participant anonymity was ensured by informing students that data would be used for research purposes only, that personal information would not be disclosed, and that unique identification codes would be used instead of names or student IDs; questionnaire data were stored separately from personal information.

Data were collected at three time points, January 2024 (T1), May 2024 (T2), and September 2024 (T3), with four-month intervals chosen for practical feasibility (alignment with university semesters) and theoretical significance (sufficient to capture short-term changes in college students’ SWB (DiLeo et al., 2022); impacts of academic and interpersonal challenges (Sun, 2023)). At T1, 505 participants provided valid data (187 male, 318 female). Attrition resulted in 23 participants lost (4 completing one wave, 19 completing two waves) because of absence, transfer, or incomplete questionnaires. The final sample comprised 482 participants completing all three waves (95.4% retention).

To assess potential attrition bias, Little’s MCAR test supported the assumption of data missing completely at random (χ^2^ = 85.46, df = 82, *p* = 0.38). Further analyses (*t*-tests, chi-square tests) revealed no significant differences between retained and attrited participants on key demographics (gender: χ^2^ = 0.31, *p* = 0.58; family ethnic composition: χ^2^ = 0.04, *p* = 0.84; ethnic minority area: χ^2^ = 0.15, *p* = 0.70) or baseline measurements of main variables (PE: t = −0.45, *p* = 0.65; EF: t = 0.53, *p* = 0.59; SWB: t = −0.34, *p* = 0.73). These findings suggest minimal attrition bias.

This study complied with the Declaration of Helsinki. Per Article 32 of China’s Ethical Review Measures for Life Science and Medical Research Involving Humans (2023), ethical review was waived for this anonymized, non-sensitive data research. The protocol received documented oversight from Xi’an Jiaotong University’s School of Journalism and New Media, with consent from all participants.

### 2.2. Measurement Instruments

Evaluation criteria for scale psychometrics were as follows: confirmatory factor analysis (CFA) with good model fit (CFI > 0.90, TLI > 0.90, RMSEA < 0.08, SRMR < 0.08) (Hu & Bentler, 1999); acceptable internal consistency (Cronbach’s α ≥ 0.70, good if ≥ 0.80) (DeVellis & Thorpe, 2021); and good test–retest reliability (correlation coefficients ≥ 0.70) (Nunnally, 1975).

#### 2.2.1. Physical Exercise

This study adapted the International Physical Activity Questionnaire (IPAQ) short form (Craig et al., 2003) to better capture exercise habituality, crucial for long-term adherence (Verplanken & Wood, 2006). Building on the IPAQ and dual-process theories, we developed a 10-item physical exercise scale with two dimensions: behavioral stability and automaticity (5-point Likert scale,1 = strongly disagree to 5 = strongly agree). The scale underwent systematic cultural adaptation (Beaton et al., 2000), including expert review (sport psychology, ethnic minority education), focus groups with ethnic minority college students (N = 12), and two pretesting rounds (N_1_ = 82, N_2_ = 97) to ensure cross-cultural applicability and content validity.

CFA indicated good structural validity across time points (χ^2^ = 145.00–155.00, df = 30–35, CFI = 0.95–0.97, TLI = 0.94–0.96, SRMR = 0.04–0.05, RMSEA = 0.04–0.06 [90% CI: 0.03–0.04, 0.08–0.09]). Factor loadings ranged from 0.84 to 0.93. Cronbach’s α for the total score was 0.88 (T1), 0.84 (T2), and 0.91 (T3), demonstrating good internal consistency. Test–retest reliability between time points was 0.84 (95% CI = [0.81, 0.92]) for T1–T2 and 0.86 (95% CI = [0.83, 0.95]) for T2–T3, indicating good scale stability.

#### 2.2.2. Executive Function

This study adapted the Behavior Rating Inventory of Executive Function (BRIEF), originally developed by Parhoon et al. (2022), for use with Chinese ethnic minority university students. Recognizing potential cultural and linguistic challenges, a cross-cultural adaptation was performed to enhance reliability and validity. Following established guidelines, the BRIEF was translated and back-translated, with discrepancies resolved to ensure semantic equivalence. The revised scale comprised 28 reverse-scored items across three dimensions, behavioral regulation (6 items), emotional regulation (8 items), and cognitive regulation (14 items), using a 5-point Likert scale (1 = strongly disagree to 5 = strongly agree).

CFA showed good structural validity across time points (χ^2^ = 158.64–182.47, df = 73–76, *p* < 0.001, CFI = 0.94–0.96, TLI = 0.93–0.95, SRMR = 0.04–0.06, RMSEA = 0.05–0.07 [90% CI: 0.04–0.06, 0.08–0.09]). Factor loadings ranged from 0.83 to 0.93. Cronbach’s α for the total score was 0.87 (T1), 0.89 (T2), and 0.91 (T3), indicating good internal consistency. Test–retest reliability between time points was 0.83 (95% CI = [0.80, 0.86]) for T1-T2 and 0.85 (95% CI = [0.81, 0.88]) for T2–T3, demonstrating good scale stability.

#### 2.2.3. Subjective Well-Being

This study adopted Magyar et al.’s definition of SWB (Magyar & Keyes, 2019), comprising life satisfaction, positive affect, and negative affect. These dimensions were measured using the Satisfaction With Life Scale (SWLS) developed by E. D. Diener et al. (1985) and the Scale of Positive and Negative Experience (SPANE) developed by E. Diener et al. (2009). A cross-cultural adaptation of the SWLS and SPANE was conducted for use with Chinese ethnic minority university students. Following established guidelines, the scales were translated and back-translated, and cultural adaptation was assessed by experts in psychology and ethnic minority languages. Item wording was modified based on expert recommendations to better align with the linguistic habits of the target population. The SWLS contains 5 items assessing overall life satisfaction. The SPANE contains 12 items (6 positive affect, 6 negative affect). All items use a 7-point scale (1 = strongly disagree to 7 = strongly agree). For the total SWB score, negative affect items are reverse-scored and then summed with life satisfaction and positive affect scores.

CFA indicated good structural validity across all time points (χ^2^ = 158.34–163.50, df = 74–77, *p* < 0.001, CFI = 0.96–0.97, TLI = 0.95–0.96, SRMR = 0.04–0.05, RMSEA = 0.06–0.07 [90% CI: 0.05–0.06, 0.08–0.09]). Factor loadings ranged from 0.87 to 0.94. Cronbach’s α was 0.90 (T1), 0.92 (T2), and 0.95 (T3), demonstrating good internal consistency. Test–retest reliability between adjacent time points was 0.86 (95% CI = [0.83, 0.89]) and 0.84 (95% CI = [0.82, 0.93]), indicating good scale stability.

### 2.3. Data Processing

Data preprocessing and descriptive statistical analyses were conducted using SPSS 26.0, including calculating means, standard deviations, and Pearson correlations for the main study variables. To examine potential baseline differences, independent sample *t*-tests (with Welch’s correction applied when necessary) were performed to assess group differences in PE, EF, and SWB across gender, family ethnic composition, and residential area. We examined these demographic variables because we hypothesized that the relationships between PE, EF, and SWB might differ based on these characteristics.

The core analysis, examining the dynamic longitudinal relationships between PE, EF, and SWB, was conducted in Mplus 8.3 in two stages:

(1) RI-CLPM: RI-CLPMs were constructed for the overall sample. This type of model estimates both within-person (changes over time) and between-person (stable individual differences) effects, providing a more accurate depiction of the dynamic relationships between variables.

(2) Multigroup analysis: To test whether gender, family ethnic composition, and residential area moderated the relationships between variables, a multigroup analysis approach was used. RI-CLPMs were estimated separately for each level of the demographic variables, and model parameters (e.g., cross-lagged effects) were compared across groups. Significant differences were considered to indicate a moderating effect.

## 3. Results

### 3.1. Common Method Bias

To mitigate common method bias (CMB), several strategies were employed. First, anonymous questionnaires were administered. Second, Harman’s single-factor test was conducted. Principal component analysis revealed that the first unrotated factor explained 18.21% (T1), 17.54% (T2), and 16.89% (T3) of the variance, all well below the 40% threshold (Podsakoff et al., 2003), suggesting minimal CMB. Furthermore, correlations between state residuals of latent variables within the RI-CLPM were estimated to control for common method variance at each time point. Finally, the longitudinal design minimized CMB through procedural separation.

### 3.2. Longitudinal Measurement Invariance

To ensure the stability of measurement structures over time, measurement invariance testing was conducted. Items from each scale were randomly parceled to optimize the parameter-to-sample ratio. Configural, metric, and scalar invariance were sequentially tested across the three time points (T1, T2, T3). Model fit was evaluated using CFI, TLI (≥0.90), RMSEA (≤0.08), and SRMR (≤0.08). Nested model comparisons were based on ΔCFI ≤ 0.01 and ΔSRMR ≤ 0.03 (Cheung & Rensvold, 2002). Results indicated that PE, EF, and SWB achieved configural, metric, and scalar invariance across the three time points (Table 1). All models exhibited acceptable fit indices (CFI and TLI > 0.90, RMSEA < 0.08 [90% CI: 0.011–0.029, 0.037–0.085], SRMR < 0.08), and acceptable ΔCFI and ΔSRMR values. These findings confirmed robust longitudinal invariance, providing a reliable basis for subsequent analyses.

### 3.3. Group Differences and Correlation Analysis

#### 3.3.1. Independent Sample *t*-Tests

Independent sample *t*-tests, with Welch’s correction for unequal variances, examined group differences in PE, EF, and SWB across gender, family ethnic composition, and residential region at T1, T2, and T3. Gender significantly affected EF and SWB (*p* < 0.05), with males scoring higher. No gender differences were found for PE (*p* > 0.05). Family ethnic composition significantly affected PE (*p* < 0.05), with interethnic families reporting higher scores. Residential region significantly influenced SWB (*p* < 0.05), with minority-concentrated areas reporting higher SWB.

#### 3.3.2. Stability and Synchrony Correlation Analysis

Descriptive statistics and partial correlation analyses were conducted for all variables, controlling for gender, family ethnic composition, and residential region (Table 2).

Stability correlations: Significant positive correlations were observed across the three measurement points for PE (rT1T2 = 0.46, rT1T3 = 0.45, rT2T3 = 0.50, *p* < 0.05), EF (rT1T2 = 0.56, rT1T3 = 0.48, rT2T3 = 0.46, *p* < 0.05), and SWB (rT1T2 = 0.48, rT1T3 = 0.46, rT2T3 = 0.50, *p* < 0.05), indicating temporal stability of these variables. Synchrony correlations: At each measurement point (T1, T2, T3), significant positive correlations were found between PE, EF, and SWB (*p* < 0.05), demonstrating synchronous relationships among the variables. These findings suggest that PE, EF, and SWB exhibited both cross-time stability and synchronous associations, providing evidence for their robust interrelations over time.

### 3.4. Between-Person-Level Analysis

Controlling for gender, family ethnic composition, and residential region, RI-CLPM was constructed to investigate the dynamic relationships among PE, EF, and SWB across three time points. The model demonstrated good fit: χ^2^ = 9.88, df = 3, RMSEA = 0.02 (90% CI = [0.01, 0.03]), CFI = 0.91, TLI = 0.94, SRMR = 0.01.

**Figure 1:**
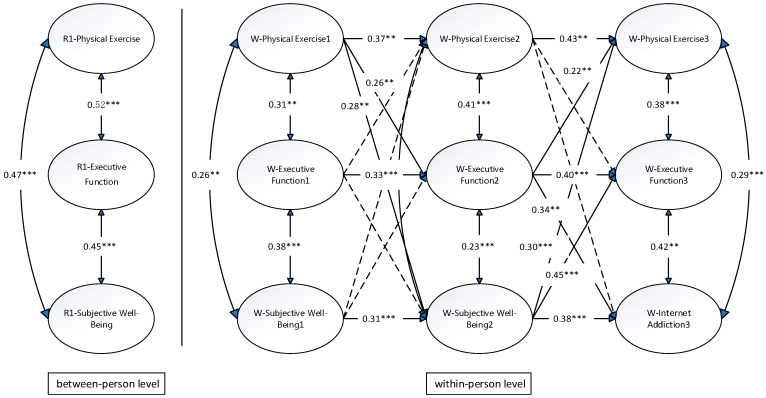
Random-intercept cross-lagged model. ** *p* < 0.01, *** *p* < 0.001.

At the between-person level, significant positive correlations were observed among PE, EF, and SWB (r range: 0.45–0.52, *p* < 0.001; see Figure 1). Specifically, PE was strongly correlated with EF (r = 0.52, *p* < 0.001) and SWB (r = 0.47, *p* < 0.001), while EF was positively associated with SWB (r = 0.45, *p* < 0.001). These findings suggest that students engaging in more frequent PE exhibited better cognitive functioning and reported higher levels of SWB. This supports the beneficial role of PE in enhancing cognitive abilities and emotional health at the interpersonal level. Consistently with Hypothesis 1, significant positive correlations were observed among PE, EF, and SWB at the between-person level, indicating that students who engaged in more frequent PE exhibited better EF and higher levels of SWB.

### 3.5. Within-Person-Level Autoregressive Results

Within-person autoregressive analyses revealed that PE, EF, and SWB maintained relatively stable developmental trajectories across adjacent time points. Significant autoregressive effects were found for all three variables, with coefficients increasing over time, indicating enhanced stability in later stages.

Specifically, baseline PE significantly predicted changes in PE at subsequent time points (β_12_ = 0.37, β_23_ = 0.43, *p* < 0.01). Similarly, EF (β_12_ = 0.33, β_23_ = 0.40, *p* < 0.001) and SWB (β_12_ = 0.31, β_23_ = 0.38, *p* < 0.001) exhibited comparable autoregressive effects. These patterns suggest cumulative effects, whereby earlier experiences exert a profound influence on later outcomes.

### 3.6. Within-Person Cross-Lagged Analysis

Results from the cross-lagged analysis indicated significant bidirectional relationships among PE, EF, and SWB at the within-person level. Specifically, PE at T1 positively predicted EF (β = 0.26, *p* < 0.01) and SWB (β = 0.28, *p* < 0.01) at T2. Additionally, EF at T2 significantly predicted PE (β = 0.22, *p* < 0.01) and SWB (β = 0.34, *p* < 0.01) at T3. Moreover, SWB at T2 positively predicted EF (β = 0.30, *p* < 0.001) and PE (β = 0.45, *p* < 0.001) at T3. These findings collectively reveal the dynamic interplay among PE, EF, and SWB, demonstrating that the three variables mutually reinforced each other, forming a self-sustaining cycle of improvement.

Further analysis of the RI-CLPM explored the strength of these causal pathways. Comparing regression coefficients, EF at T2 exerted a stronger influence on SWB at T3 (β = 0.34) than SWB at T2 did on EF at T3 (β = 0.30). This highlights the pivotal role of EF within the dynamic system.

Consistently with Hypothesis 2, the within-person autoregressive and cross-lagged analyses collectively revealed significant dynamic reciprocal predictive relationships among PE, EF, and SWB, indicating their mutual reinforcement and cumulative effects over time.

### 3.7. Multiple Group Analysis

To examine whether the dynamic relationships among PE, EF, and SWB were moderated by gender, family structure, and residential region, this study employed MGA using RI-CLPM and the grouping syntax in Mplus 8.3. The influence of group variables on intervariable interactions was evaluated by comparing the fit of freely estimated models and models with cross-group equality constraints on path coefficients.

For the gender-based analysis, the freely estimated model showed excellent fit: χ^2^ = 0.57, df = 3, χ^2^/df = 0.19, CFI = 1.00, TLI = 1.02, SRMR = 0.02, RMSEA = 0.01 (90% CI = [0.005, 0.024]). The TLI value of 1.02, exceeding the conventional upper limit of 1.0, indicated an exceptionally good fit between the model and the data. The very low chi-square value (χ^2^ = 0.57) with small degrees of freedom (df = 3) further supported this, suggesting that the model almost perfectly reproduced the observed covariance matrix. The equality-constrained model also demonstrated good fit: χ^2^ = 13.24, df = 12, χ^2^/df = 1.10, CFI = 0.99, TLI = 0.98, SRMR = 0.05, RMSEA = 0.03 (90% CI = [0.02, 0.04]). A chi-square difference test revealed no significant difference between the two models (Δχ^2^(9) = 12.67, *p* = 0.176, *p* > 0.05), indicating that gender did not moderate the cross-lagged paths or random-intercept relationships among the variables.

Parallel analyses for family structure and residential region similarly showed no significant differences between freely estimated and equality-constrained models: for family structure (Δχ^2^(10) = 10.94, *p* = 0.293) and residential region (Δχ^2^(8) = 11.85, *p* = 0.184). Both model types consistently exhibited robust fit indices, confirming consistency in the cross-lagged pathways and random intercept correlations across these subgroup contexts.

Contrarily to Hypothesis 3, MGA revealed that gender, family ethnicity, and residential location did not significantly moderate the dynamic relationships among PE, EF, and SWB, suggesting that these interaction mechanisms are stable and invariant across different sociodemographic groups.

## 4. Discussion

This study, employing RI-CLPM and MGA, systematically examined the cross-lagged pathways and dynamic interaction mechanisms among PE, EF, and SWB across three waves of measurement data in a sample of minority college students. Consistently with Hypotheses 1 and 2, our comprehensive analyses revealed significant positive direct and dynamic relationships among PE, EF, and SWB, highlighting PE’s role in promoting both cognitive and emotional functions, and demonstrating the spatiotemporal stability and cumulative effects of these interconnections. Conversely, Hypothesis 3, which proposed that these dynamic relationships would be moderated by sociodemographic factors, was not supported; instead, the study found a remarkable consistency in cross-lagged pathways across gender, family ethnic composition, and residential region. These results not only expand existing dynamic interaction theories but provide practical implications for individualized psychological health interventions. The discussion is structured around group-specific effects, the robustness of interaction mechanisms, and their theoretical and practical significance.

### 4.1. Group Effects of Gender, Family Structure, and Residential Region on PE, EF, and SWB

MGA revealed nuanced effects of gender, family ethnic composition, and residential region on the study variables, enriching our understanding of the interplay between sociodemographic contexts and individual well-being.

Gender exerted a significant influence on both EF and SWB, with male participants demonstrating higher scores in these dimensions. This finding is congruent with existing literature, suggesting that gender-linked neurocognitive variations may contribute to differential EF performance (Grissom & Reyes, 2019). Specifically, males often exhibit enhanced capabilities in cognitive domains such as spatial reasoning and processing speed, both pivotal for EF. Furthermore, the observed gender disparity in SWB may be attributable to variations in socialization patterns and coping mechanisms. Males are often socialized to externalize emotions and adopt problem-focused coping strategies, whereas females may be more inclined towards internalizing stress, potentially impacting their overall SWB (Batz-Barbarich et al., 2018). It is noteworthy that no significant gender differences emerged in PE, intimating that engagement in PE may be governed by shared motivational factors and environmental affordances rather than being solely determined by gender.

Family ethnic composition and residential region uniquely influenced PE and SWB, respectively. The association between interethnic family backgrounds and elevated levels of PE suggests that physical activity may serve as a salient conduit for cross-cultural interaction and social integration. Interethnic families often navigate a mosaic of cultural norms and values, which may cultivate a heightened appreciation for physical activity as a vehicle for promoting health and social cohesion. Conversely, the elevated levels of SWB reported by individuals residing in minority-concentrated areas underscore the critical role of cultural identity, social connectedness, and communal support in fostering emotional well-being. Minority communities frequently foster robust social networks and a pronounced sense of belonging, which may buffer against the deleterious effects of stress and discrimination (Darvishmotevali et al., 2018; Gong et al., 2019). These findings highlight the imperative of considering the broader sociocultural milieu when examining individual-level outcomes.

The invariance observed in the cross-lagged pathways and random intercept correlations among PE, EF, and SWB across gender, family ethnic composition, and residential region, as evinced by the MGA, furnishes compelling evidence for the robustness of the underlying dynamic relationships. This suggests that the fundamental mechanisms interlinking physical activity, cognitive functioning, and emotional well-being remain consistent across diverse sociodemographic strata, thereby reinforcing the potential of PE as a universally beneficial intervention modality. However, it remains crucial to acknowledge that while the relationships exhibited invariance, the levels of PE, EF, and SWB may diverge across groups, underscoring the importance of tailoring interventions to address context-specific needs and cultural nuances. For instance, interventions designed to augment PE in interethnic families could focus on leveraging culturally relevant activities and bolstering social support networks, whereas interventions aimed at ameliorating SWB in minority communities could emphasize the cultivation of social cohesion and cultural pride.

### 4.2. Interindividual Effects and Theoretical Extension

At the individual level, our study observed significant positive correlations among PE, EF, and SWB in the sample of minority college students. These results indicate that even after controlling for gender, family ethnic composition, and residential region, minority college students who engaged in more frequent PE tended to exhibit better cognitive function and report higher levels of SWB.

This study confirmed the role of PE in enhancing EF and SWB at the group level, aligning with existing theories in cognitive neuroscience and psychology. Previous research has demonstrated that PE improves EF—encompassing working memory and attentional control—by enhancing cerebral blood flow and activity in the prefrontal cortex (Hashimoto et al., 2018). Moreover, exercise promotes emotional health by modulating the release of neurotransmitters such as endorphins, effectively reducing anxiety and increasing happiness (Kleinloog et al., 2019). By examining these phenomena at the level of individual differences, this study further extends these findings, highlighting the stability and universality of the psychological effects of exercise across different individuals.

Minority college students, as a group facing significant cultural adaptation stress, demonstrate a unique amplification of the positive effects of PE. Cultural adaptation stress arises from challenges in adjusting to a new cultural environment, manifesting as feelings of discrimination, social isolation, and pressure to conform (Kim & Kim, 2019). These stressors can adversely affect cognitive function and mental health, making effective coping mechanisms especially important for this population. Our findings indicate that PE significantly enhanced the EF and SWB of this group, suggesting that physical activity is not only an effective individual-level intervention but, when combined with culturally sensitive or traditional forms of exercise, can facilitate cultural exchange and social cohesion. One potential mechanism through which exercise exerts these beneficial effects is by increasing levels of BDNF, a protein that supports neuronal growth and survival, which has been shown to decrease under stress (Notaras & van den Buuse, 2020). Additionally, engaging in physical activities within supportive social environments (such as culturally relevant sports teams) can alleviate feelings of isolation and promote a sense of belonging, further enhancing SWB.

While this study provides valuable insights, it is important to acknowledge certain limitations. The reliance on self-reported measures may introduce potential biases, and the generalizability of the findings may be limited to a similar population of minority college students.

Given these findings, interventions aimed at promoting physical activity among minority college students may represent a promising strategy for enhancing cognitive and emotional well-being. However, it is crucial that these interventions are culturally tailored to meet the specific needs and preferences of the target population. For example, incorporating traditional sports or dance forms familiar to minority students may be more effective than generic exercise programs. Furthermore, interventions should address potential barriers to PE, such as a lack of adequate facilities or culturally appropriate activities. By designing culturally sensitive and effective interventions, we can maximize the potential benefits of PE for minority college students. Future research should explore the specific mechanisms through which exercise affects the EF and SWB of this population, as well as the potential moderating roles of cultural identity, social support, and cultural adaptation stress.

### 4.3. Within-Person Cumulative Effects: Long-Term Synergistic Role of PE

At the within-person level, this study revealed the temporal stability and cumulative effects among PE, EF, and SWB. The findings suggest that early engagement in PE not only yields short-term cognitive and emotional benefits but accumulates over time to form a sustainable positive cycle. This supports core principles in developmental psychology and behavioral intervention theories, which emphasize that early positive behavioral choices can have long-term impacts on the developmental trajectories of psychological variables (Gardner & Lally, 2023). The “snowball effect” of positive behaviors underscores the importance of early intervention and the potential for long-term gains.

The cumulative effects highlight the temporal sensitivity and synergistic mechanisms of PE in promoting EF and SWB. Prior research has noted that EF not only shapes information processing and behavioral control but exerts cumulative influence on emotional regulation and social interactions over time (Farroni et al., 2022; Q. Li et al., 2020). Individuals with stronger EF skills are better equipped to manage stress, regulate emotions, and foster positive relationships, all of which contribute to enhanced well-being. This study further indicates that PE directly facilitates the development of EF, and EF, in turn, modulates trends in physical activity engagement and contributes to feedback loops that amplify SWB. As individuals experience the positive impacts of physical activity on their cognitive and emotional health, they are more likely to continue these behaviors, thus creating a virtuous cycle. Collectively, these findings suggest that PE, EF, and SWB operate as a dynamically interconnected system that evolves synergistically over time.

The cumulative effects are particularly significant for minority college students, who face prolonged psychological stress associated with linguistic, cultural, and academic adaptation. PE intervention not only serves as a short-term stress buffer but establishes long-term synergistic effects with EF and SWB, thereby fostering enduring psychological resilience. By strengthening EF skills, PE can help minority students better navigate the challenges of acculturation and academic life, leading to improved emotional health and enhanced self-efficacy. These findings underscore the necessity of designing interventions that move beyond short-term measures to focus on systematic, long-term development. For example, implementing continuous PE programs can indirectly enhance SWB by strengthening EF, thereby creating a stable positive developmental cycle. The design of these programs should be culturally sensitive and inclusive of all students, taking into account their unique needs and experiences.

### 4.4. Bidirectional Dynamic Interaction Mechanism and the Central Role of EF

The cross-lagged analysis in this study revealed the bidirectional interaction mechanism among PE, EF, and SWB, with particular emphasis on the central role of EF within the psychological dynamic system. This finding underscores the pivotal role of EF in regulating psychological variables, including its influence on behavioral choices and the organization of higher-order cognitive functions (Poon, 2018). EF encompasses a range of cognitive skills, such as planning, working memory, and inhibitory control, which are critical for goal-directed behavior and adaptive functioning. The results demonstrated that EF not only significantly predicts future PE engagement and changes in SWB but fosters a dynamic feedback loop through behavioral proactivity and emotional regulation. Individuals with stronger EF skills are more likely to plan and initiate physical activities, persevere when facing challenges, and regulate emotions to cope with stress, all of which contribute to a positive cycle of well-being.

The reverse pathways of SWB are equally noteworthy. This study found that SWB is not merely an outcome variable of cognitive and behavioral processes but actively contributes to the optimization of cognitive functions and the expansion of behavioral engagement through positive emotional effects. This confirms the conclusions of previous research, where positive emotions can broaden individuals’ attention span, enhance creativity, and improve problem-solving abilities (Dinius et al., 2023). For example, individuals with higher levels of SWB exhibit greater cognitive flexibility and enhanced decision-making abilities, which further amplify the benefits of PE. They may be more willing to try new activities, more resilient when facing setbacks, and more likely to seek social support, all of which contribute to a more positive and fulfilling lifestyle. These findings highlight the reciprocal facilitation among PE, EF, and SWB in the temporal dynamic model, providing robust evidence for the bidirectional relationships underpinning psychological variables.

This bidirectional mechanism is particularly relevant for minority college students, who experience heightened cultural adaptation stress. The regulatory role of EF in this population not only significantly affects behavioral habits and SWB levels but improves the interaction dynamics among psychological variables through reciprocal pathways. By strengthening EF skills, minority students can better manage the challenges of cultural adaptation, regulate emotions, and adopt proactive coping strategies. Practical interventions could integrate cognitive and emotional training, such as culturally inclusive physical activities combined with EF-enhancing tasks (e.g., mindfulness exercises, cognitive restructuring), to strengthen individuals’ cognitive regulation capacity and ultimately achieve long-term optimization of SWB. The design of these interventions should be accessible, culturally sensitive, and integrated into existing support systems for minority students.

In conclusion, this study validated the bidirectional facilitative effects among PE, EF, and SWB using a dynamic interaction model, offering substantial theoretical and practical contributions to psychology, education, and sociology. Beyond enriching the theoretical discourse through the integration of cultural adaptation theory, cognitive neuroscience, and positive psychology to understand PE–EF–SWB interactions in a multistress context, this research addresses a critical gap by focusing on the unique and underserved population of Chinese ethnic minority college students. Furthermore, the methodological rigor, particularly the application of the advanced RI-CLPM to three-wave longitudinal data, significantly strengthens the potential for causal inference, effectively controlling for initial individual differences and overcoming the limitations of traditional cross-sectional studies. These findings not only provide critical guidance for improving psychological intervention policies but carry profound implications for the development of mental health support systems in multiethnic societies worldwide. By promoting physical activity, cultivating cognitive skills, and fostering emotional well-being, we can create more resilient and flourishing communities for all.

## 5. Conclusions

This study employed an RI-CLPM and multigroup analysis to systematically and thoroughly investigate the dynamic, time-evolving interplay between PE, EF, and SWB within a sample of Chinese ethnic minority college students. The main findings clearly revealed significant, bidirectional, and positive longitudinal relationships among PE, EF, and SWB, highlighting the cumulative, beneficial effects of PE on both cognitive function and emotional well-being over time. Notably, EF not only played a significant bridging role in mediating the positive influence of PE on SWB but occupied a central pivotal position within this dynamic system, forming a tightly interconnected network with the other variables. Critically, these complex dynamic pathways exhibited a high degree of consistency across subgroups defined by gender, family ethnic composition, and residential area, confirming the generalizability and robustness of the hypothesized interactive model within the studied population.

### 5.1. Theoretical Implications

This study provides critical new evidence for a dynamic, interactive theoretical framework linking PE, EF, and SWB, successfully constructing and validating a bidirectional interactive model unfolding across time. The findings profoundly reveal that PE not only directly influences individual psychological functioning as a behavioral intervention but, more importantly, forms a closed-loop mechanism characterized by feedback and reinforcement through dynamic interactive pathways with other core psychological variables (EF and SWB). This theoretical model not only confirms the bidirectional, reciprocal relationships among PE, EF, and SWB but challenges potentially linear or unidirectional causal assumptions inherent in traditional psychological research, emphasizing the importance of understanding the complexity, reciprocity, and feedback mechanisms within psychological variables. Furthermore, the identification of EF as a central pivotal hub within this dynamic system, along with its multifaceted connectivity, reinforces its core importance in cognitive regulation and the integrated development of overall psychological functioning, providing a solid empirical foundation for future explorations of theoretical models integrating cognitive, emotional, and behavioral development. Concurrently, the confirmation of reciprocal influence pathways of SWB within the dynamic interactive model expands the theoretical horizons and methodological avenues for future research involving more complex, multivariate dynamic systems models.

### 5.2. Practical Insights

From a practical application perspective, the findings of this study offer valuable guidance for promoting the psychological well-being and cultural adaptation of Chinese ethnic minority college students. First, given that PE serves as a low-cost, easily disseminated, and highly universal behavioral intervention, it should be systematically integrated into long-term psychological support systems targeting this population, with an emphasis on sustained implementation to maximize its cumulative positive effects on EF and SWB. At a concrete operational level, designing and promoting culturally sensitive physical activities (e.g., incorporating traditional ethnic sports elements or projects aligned with local cultural preferences) can not only significantly enhance the acceptance and participation rates of interventions but contribute to strengthening individual cultural identity and social adaptability. Second, this study suggests that EF training should be considered an important complementary component of psychological health intervention programs. By specifically enhancing individual cognitive control abilities (e.g., through multitasking exercises, inhibitory control tasks, or working memory training), one can indirectly optimize behavioral regulation strategies, thereby improving SWB. Finally, the universality of the PE intervention identified in this study, and its positive impact on EF and SWB, holds broad promotional value within a multiethnic national context. Particularly in groups facing significant cultural differences and adaptation challenges, physical activity not only effectively improves individual psychological states but has the potential to serve as a crucial bridge for promoting intergroup understanding, cooperation, and social integration, contributing to the construction of a harmonious and inclusive multicultural society.

## 6. Limitations and Future Research Directions

Despite its significant contributions to the construction of a dynamic interaction model, this study has several limitations. First, the sample was drawn exclusively from Chinese minority college students, limiting the generalizability of the findings to other cultural contexts. Future studies should expand the sample to include participants from diverse multicultural backgrounds to examine whether similar interaction mechanisms exist across different populations. Second, this study did not differentiate PE by type or intensity, leaving unexplored the extent to which various forms of physical activity contribute to the interaction among PE, EF, and SWB. Future research could employ experimental designs to evaluate intervention effects across different types of physical activities (e.g., aerobic exercise vs. resistance training). Finally, the moderating role of social factors, such as family background and social support, in this interaction mechanism requires further investigation. Understanding these contextual factors could provide deeper insights into how dynamic processes are shaped by broader social environments.

## Figures and Tables

**Table 1:** Longitudinal measurement invariance test for each variable.

Variable	Model	χ^2^/df	CFI	TLI	SRMR	RMSEA	Model Comparison	ΔCFI	ΔSRMR
Physical Exercise	M1	2.90	0.987	0.961	0.043	0.057			
	M2	2.11	0.983	0.983	0.045	0.052	M2—M1	−0.004	0.002
	M3	1.19	0.980	0.975	0.047	0.054	M3—M2	−0.003	0.002
Executive Function	M1	2.50	0.988	0.979	0.036	0.034			
	M2	1.40	0.989	0.976	0.037	0.045	M2—M1	0.001	0.001
	M3	1.24	0.981	0.970	0.041	0.028	M3—M2	−0.008	0.004
Subjective Well-Being	M1	1.25	0.927	0.969	0.048	0.047			
	M2	1.35	0.925	0.963	0.051	0.055	M2—M1	−0.002	0.003
	M3	1.38	0.922	0.943	0.052	0.038	M3—M2	−0.003	0.001

**Table 2:** Descriptive statistics and correlation analysis results for physical exercise, executive function, and subjective well-being.

Variable	M	SD	1	2	3	4	5	6	7	8	9	10	11	12
1. Gender	1.63	0.48	1											
2. Minority Region	1.40	0.49	0.20 *	1										
3. Interethnic Marriage	1.70	0.46	0.11 *	0.15 *	1									
4. T1 Physical Exercise	14.24	2.34	0.35 **	0.45 **	0.15 *	1								
5. T2 Physical Exercise	15.24	1.18	0.32 **	0.42 **	0.18 *	0.46 **	1							
6. T3 Physical Exercise	14.18	2.21	0.30 **	0.41 **	0.17 *	0.45 **	0.50 **	1						
7. T1 Executive Function	13.27	2.48	0.19 *	0.20 *	0.10	0.28 *	0.35 *	0.33 *	1					
8. T2 Executive Function	14.28	3.04	0.22 *	0.22 *	0.11	0.30 *	0.38 *	0.36 *	0.56 **	1				
9. T3 Executive Function	14.49	4.04	0.21 *	0.23 *	0.12	0.33 *	0.36 *	0.34 *	0.48 **	0.46 **	1			
10. T1 Subjective Well-Being	55.37	8.21	0.25 *	0.30 *	0.13	0.40 *	0.43 *	0.42 *	0.44 *	0.40 *	0.36 *	1		
11. T2 Subjective Well-Being	58.14	5.58	0.28 *	0.29 *	0.12	0.38 *	0.42 *	0.41 *	0.47 *	0.42 *	0.38 *	0.48 **	1	
12. T3 Subjective Well-Being	57.27	6.29	0.27 *	0.31 *	0.15	0.36 *	0.41 *	0.40 *	0.45 *	0.41 *	0.35 *	0.46 **	0.50 **	1

Note: * *p* < 0.05, ** *p* < 0.01.

## Data Availability

The datasets analyzed in this study are available from the corresponding author upon reasonable request.
